# Within- and between-field variability in natural turfgrass and synthetic turf is associated with differences in athlete mechanical loading and perception

**DOI:** 10.3389/fspor.2026.1876412

**Published:** 2026-06-22

**Authors:** Ava Veith, David McCall, Daniel Sandor, Chase Straw, Jay Williams, John H. Challis

**Affiliations:** 1 School of Plant and Environmental Sciences, Virginia Tech, Blacksburg, VA, United States; 2 Department of Plant Science, Pennsylvania State University, University Park, PA, United States; 3 Department of Human Nutrition, Foods, and Exercise, Virginia Tech, Blacksburg, VA, United States; 4 Biomechanics Laboratory, Pennsylvania State University, University Park, PA, United States

**Keywords:** athlete-surface interactions, athletic fields, female athlete, inertial measurement units (IMUs), natural turfgrass, synthetic turf

## Abstract

**Introduction:**

Natural turfgrass and synthetic turf athletic fields exhibit variability within and between surface types due to factors such as usage and maintenance, yet limited research has examined how this variability is associated with differences in athlete mechanical loading and perception.

**Methods:**

This study quantified surface characteristics and athlete responses across four athletic fields, two natural turfgrass and two synthetic turf, with one high-usage and one low-usage field of each type. Surface hardness, rotational resistance, infill depth, thatch depth, and soil moisture were evaluated to characterize field conditions. Fourteen female athletes completed standardized drills while wearing Blue Trident ankle inertial measurement units (IMUs) and STATSports APEX GPS performance trackers to quantify lower-limb impact loading and running speed, respectively. Athlete perceptions of surface conditions were assessed using pre- and post-performance surveys.

**Results and Discussion:**

Surface hardness varied significantly within and between field types (*p* < 0.001), with mean values of 61.3 and 69.7 Gmax for low- and high-usage synthetic turf, respectively, and 46.8 and 49.8 Gmax for low- and high-usage natural turfgrass. Harder surfaces, particularly synthetic turf, resulted in greater lower-limb impact intensity, increasing from an average intensity of 17.8 g on natural turfgrass to 20.7 g on synthetic turf across drills. Within synthetic turf fields, certain higher-hardness areas produced “high-intensity” ankle IMU classifications, indicating increased mechanical loading in these regions. Survey results indicated that athletes rated the low-usage natural turfgrass field most positively, reporting higher surface quality and less negative impact on performance, while both synthetic turf fields received more positive ratings than the high-usage natural turfgrass field. Overall, results demonstrate that spatial and between-field variability in surface properties can influence athlete loading and perception, highlighting the importance of considering both objective surface characteristics and athlete experience when evaluating athletic field safety and performance.

## 1 Introduction

Athletic playing fields represent a modifiable environmental factor that may impact athlete biomechanics and injury risk. Extensive research has compared injury rates, biomechanics, and athlete perceptions between natural turfgrass and synthetic turf surfaces (1–3). However, many investigations categorize fields broadly as “natural turfgrass” or “synthetic turf,” overlooking within-field variability that may meaningfully impact athlete-surface interactions (4–6). Even when surfaces meet governing body standards, localized differences in surface properties can alter impact transmission to the lower limbs and influence movement mechanics during sport-specific tasks (7, 8).

Surface characteristics such as hardness (9), rotational traction or resistance (10, 11), soil moisture (12, 13), thatch depth (14), and infill depth (15) vary spatially within both natural turfgrass and synthetic turf systems and directly influence energy dissipation and traction. As a result, these properties may contribute to differences in lower-limb mechanical loading, yet the role of within-field variability in shaping athlete biomechanics remains insufficiently understood (16, 17).

Wearable technologies have become central to quantifying movement demands and training loads (18). Global positioning systems (GPS) and inertial measurement units (IMUs) enable field-based monitoring of variables such as trunk acceleration, running speed, and lower-limb impact loading (19, 20). The STATSports APEX Athlete Series GPS (STATSports, Newry, Northern Ireland, UK) provides commonly used trunk acceleration and speed metrics with demonstrated reliability (21–24). Trunk acceleration is relevant to anterior cruciate ligament (ACL) injury mechanisms, as lateral trunk motion increases knee abduction loading (25–28). Female athletes experience approximately 3.5-fold higher risk of non-contact ACL injury compared with male athletes (29). Evaluating how surface conditions influence trunk and lower-limb loading in female athletes warrants further investigation. Ankle-worn IMUs, such as the IMeasureU Blue Trident system (Blue Trident, IMeasureU, Auckland, New Zealand), quantify cumulative impact metrics including average intensity, impact load, and bone stimulus, providing estimates of lower-limb mechanical loading during athletic movement (19). These metrics offer a practical approach to evaluating how within- and between-field variability in surface characteristics may influence athlete loading responses in field settings (16). While previous work has documented variability in surface characteristics (4–6), direct relationships between this variability and athlete-derived biomechanical loading remain limited (5).

In addition to mechanical loading, athlete perception may influence movement strategy and performance. Variability within natural turfgrass fields can alter perceived stability and confidence during play (30), potentially affecting movement behavior and decision-making (31). Integrating objective evaluations of surface properties with biomechanical loading metrics and athlete perception could provide a comprehensive understanding of athlete–surface interactions.

Taken together, these findings suggest that athletic fields should not be viewed simply as natural turfgrass or synthetic turf surfaces, but rather as environments that exhibit meaningful spatial variability in their mechanical properties. Variability in surface characteristics such as hardness, rotational resistance, soil moisture, thatch depth, and infill depth can create localized differences in the mechanical environment experienced by athletes, which may influence mechanical loading, performance, and subjective perceptions of surface quality. Because field-wide averages may not fully represent the range of conditions encountered during play, integrating objective measurements of surface characteristics with athlete-derived biomechanical and perceptual responses may provide a more comprehensive understanding of athlete–surface interactions and the implications of within- and between-field variability for athletic field evaluation and management.The objective of this study was to quantify how variability in surface characteristics across natural turfgrass and synthetic turf athletic fields influences athlete mechanical loading, performance metrics, and perception, considering differences both within and between surface types. It was hypothesized that 1) surface characteristics would vary both within and between field types, resulting in localized differences in the environments experienced by athletes; (2) these differences would be reflected in athlete biomechanical responses, with firmer surface characteristics associated with greater IMU-derived loading metrics; and (3) athlete perceptions of surface quality would correspond with objectively measured differences in surface characteristics.

## 2 Materials and methods

### 2.1 Field locations

**Figure 1:**
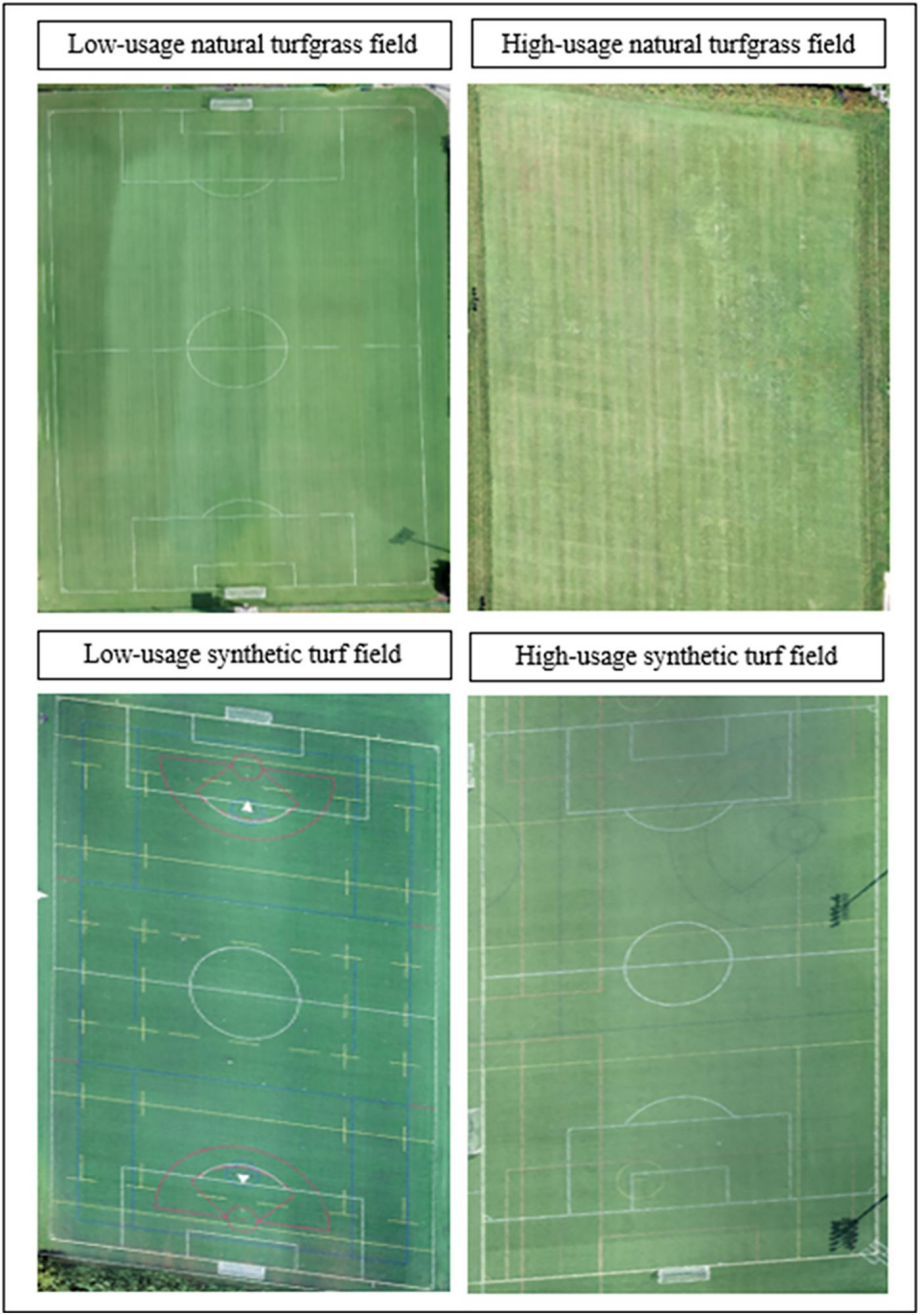
Aerial (drone) imagery of the four athletic fields used in this study, including low- and high-usage natural turfgrass fields (top row) and low- and high-usage synthetic turf fields (bottom row) located at Virginia Polytechnic Institute and State University (Blacksburg, VA, USA). Images illustrate differences in surface condition and usage intensity across field types.

Data collection was conducted on 19–20 August 2024 at four athletic fields on the Virginia Polytechnic Institute and State University (Virginia Tech) campus in Blacksburg, Virginia. Two fields were natural turfgrass, and two were synthetic turf. Fields were classified *a priori* by surface type and relative usage (low and high) based on typical traffic and event frequency (Table 1; Figure 1). Field usage was included as a factor because traffic intensity can influence athletic field surface conditions. Categorizing fields as high- or low-usage allowed evaluation of whether athlete responses differed not only by surface type, but also by the level of field use. This distinction reflects a common management scenario in which fields of the same surface type may experience substantially different levels of traffic.

**Table 1:** Characteristics of four athletic fields located at Virginia Polytechnic Institute and State University (Blacksburg, VA, USA) evaluated in August 2024, including field type, bermudagrass cultivar or fiber type, mowing height or fiber length, soil or infill composition, usage intensity, and study classification.

Field Type	Field	Hybrid Bermudagrass Cultivar/Fiber Type	Mowing Height/Fiber Length	Soil or Infill Type	Usage Per Week	Study Classification
Natural Turfgrass	Soccer & Lacrosse Stadium Field	Patriot (primary), Tahoma 31 (goalmouths), Latitude 36 (center)	15.9 mm	Native soil (highly amended with 20 years of sand topdressing)	∼2 × per week (spring/summer); ∼5 times per week (fall);	Low-usage natural turfgrass field
Natural Turfgrass	Rugby Practice Field	Latitude 36 (sprigged with Tahoma 31)	25.4 mm	Native soil	∼5 times per week (spring/fall); open access daily	High-usage natural turfgrass field
Synthetic Turf	Club Sports Synthetic Turf Field	Slit-film and monofilament blend	63.5 mm	Sand & crumb rubber mixture	∼6 times per week (spring/fall); open access daily	Low-usage synthetic turf field
Synthetic Turf	Recreational Synthetic Turf Field	Slit-film and monofilament blend	63.5 mm	Sand & crumb rubber mixture	∼6 times per week year-round; open access daily	High-usage synthetic turf field

The low-usage natural turfgrass field was constructed in 2003 and is currently a hybrid bermudagrass [*Cynodon dactylon* (L.) Pers. × *Cynodon transvaalensis* Burtt-Davy] surface consisting of the cultivars “Patriot”, “Tahoma 31”, and “Latitude 36”, maintained to meet National Collegiate Athletic Association (NCAA) Division I requirements. The field consists of native soil that has been amended with approximately 20 years of sand topdressing and is equipped with a Cambridge drainage system (32).

The high-usage natural turfgrass field was a rugby practice field established on native soil and consisting primarily of “Latitude 36” hybrid bermudagrass, with “Tahoma 31” introduced through localized re-sprigging in the summer of 2023 to aid recovery of areas affected by wear and winter injury. This field supported frequent practices, competitions, and recreational use and received less intensive routine maintenance due to labor and budget constraints.

The low-usage synthetic field was installed in summer 2023 and used primarily by Virginia Tech club sports. The high-usage synthetic field was installed in 2015 within a multi-field recreational complex and experienced frequent multi-sport use and public access. Both fields consisted of FieldTurf (FieldTurf Turf Systems Classic HD; Calhoun, GA), contained a sand and crumb rubber infill mixture, were groomed approximately every six weeks, swept for debris as needed, and did not include a shock pad (Table 1).

### 2.2 Surface hardness interpolation maps

Surface hardness was selected due to its established relationships with athlete performance and surface response characteristics (33, 34), and mapping was completed two weeks prior to athlete testing to identify relatively hard and soft testing areas for subsequent testing. 100 surface hardness measurements were collected at each of the four field locations using a PNCLEGG-S-2.25-A Clegg Impact Soil Tester equipped with a 2.25 kg hammer and flat end (Turf-Tec International, Tallahassee, FL). The hammer was dropped from a fixed height of 45 cm, and calibration was verified prior to sampling using the manufacturer-provided pad. Hardness data (Gmax) were recorded using a Samsung Galaxy Tablet “A” (San Jose, CA 95134) with the Clegg Control application (v1.12, Lafayette Instruments Company, Inc.).

Sampling locations were arranged as a 10 × 10 grid, resulting in 100 evenly distributed points across each field. Because field dimensions varied slightly, spacing between points was determined by dividing field length and width into equal segments, with sampling conducted at each intersection. One Clegg drop was performed at each sampling point, and GPS coordinates were collected concurrently at each location using an Emlid Reach rover and base system (Emlid Tech Kft., Budapest, Hungary) to enable real-time kinematic correction. These georeferenced data were used to generate spatial surface hardness maps.

Georeferenced data were interpolated in ArcGIS Pro version 2.7.1 (ESRI, Redmond, California) using inverse distance weighting (power = 2; 12 neighboring points), and an optimized hot spot analysis was used to identify statistically distinct high- and low-hardness regions within each field. These interpolated maps were used to delineate two rectangular testing areas per field representing relatively higher and lower hardness values. Hard and soft areas were defined relative to the distribution of surface hardness within each field and therefore represent within-field contrasts rather than absolute hardness categories across fields. Surface hardness data (*n* = 100 per field) were analyzed using one-way analysis of variance, with means separated using Fisher's protected least significant difference (LSD) test at *α* = 0.05. Data were analyzed in JMP Pro 2024 version 18.0.0 (SAS Institute Inc., Cary, NC).

### 2.3 Field properties within selected hard and soft areas

Additional evaluations were conducted within the designated hard and soft areas of each field prior to athlete arrival on the day of testing to further characterize surface conditions. On natural turfgrass fields, rotational resistance, thatch depth (the organic layer of living and dead plant material between the turf canopy and soil surface), and soil moisture were measured, whereas on the synthetic turf fields, rotational resistance and infill depth were measured. Twenty measurements per area were collected for each metric.

Rotational resistance was evaluated using the Deltec rotational resistance tester (Deltec, Duiven, The Netherlands), thatch depth (mm) using a soil profile sampler (MPS2-S Mascaro, Turf-Tec International, Tallahassee, Florida), soil moisture (percent volumetric water content) using a TDR 350 Soil Moisture Meter (Spectrum Technologies, Inc., Aurora, Illinois) with 7.6 cm tines, and infill depth using a Turf-Tec Professional Model Infill Depth Gauge (Turf-Tec International, Tallahassee, Florida). Measurements were collected in a randomized pattern within each testing area and averaged to characterize conditions representative of each area.

For rotational resistance, soil moisture, thatch depth, and infill depth data, analyses were conducted using a standard least squares model in JMP Pro 2024, with field type (natural turfgrass or synthetic turf), field usage (high or low), and hardness (hard or soft within-field selected area) treated as fixed effects. Two- and three-way interactions among these factors were initially included but were not statistically significant and did not improve model fit; therefore, a reduced model including only main effects was retained. Significant main effects were interpreted using effect tests, and mean values were used to describe within-field variability between hard and soft areas.

### 2.4 Athlete performance

Approval to conduct human subjects research was granted by the Virginia Polytechnic Institute and State University Institutional Review Board [IRB#24-395]. All participants provided written informed consent prior to participation. A total of fourteen female athletes were recruited. Participants' mean (± SD) age, mass, height, and years of athletic experience were 18.6 ± 2.1 years, 64.9 ± 6.8 kg, 166.9 ± 7.2 cm, and 10.8 ± 3.3 years, respectively. All participants reported no prior or existing injuries and were healthy throughout the study. Participants had previous competitive athletic experience in field-based sports and were familiar with the sprinting, jumping, deceleration, and change-of-direction movements included in the testing protocol. Data collection occurred on 19–20 August 2024. On 19 August, athletes completed testing on the two synthetic turf fields, and on 20 August on the two natural turfgrass fields. Participants were randomly assigned to two groups of seven, with sessions from approximately 12:30–14:30 and 15:00–17:00. These time periods were selected to allow for surface condition measurements prior to athlete arrival, completion of all testing procedures within a single session, and preparation between groups. The selected hard and soft areas within each field were intentionally large enough to ensure that testing locations could be spatially distributed, minimizing the likelihood that surface conditions in the later session were influenced by athlete traffic from the earlier session. Participant group membership remained consistent across both testing days, and the order of field testing was predetermined and consistent across participants. Within each field, however, athletes were randomly assigned to begin testing in either the designated hard or soft area before crossing over to the opposite area after completing all drills and surveys.

**Figure 2:**
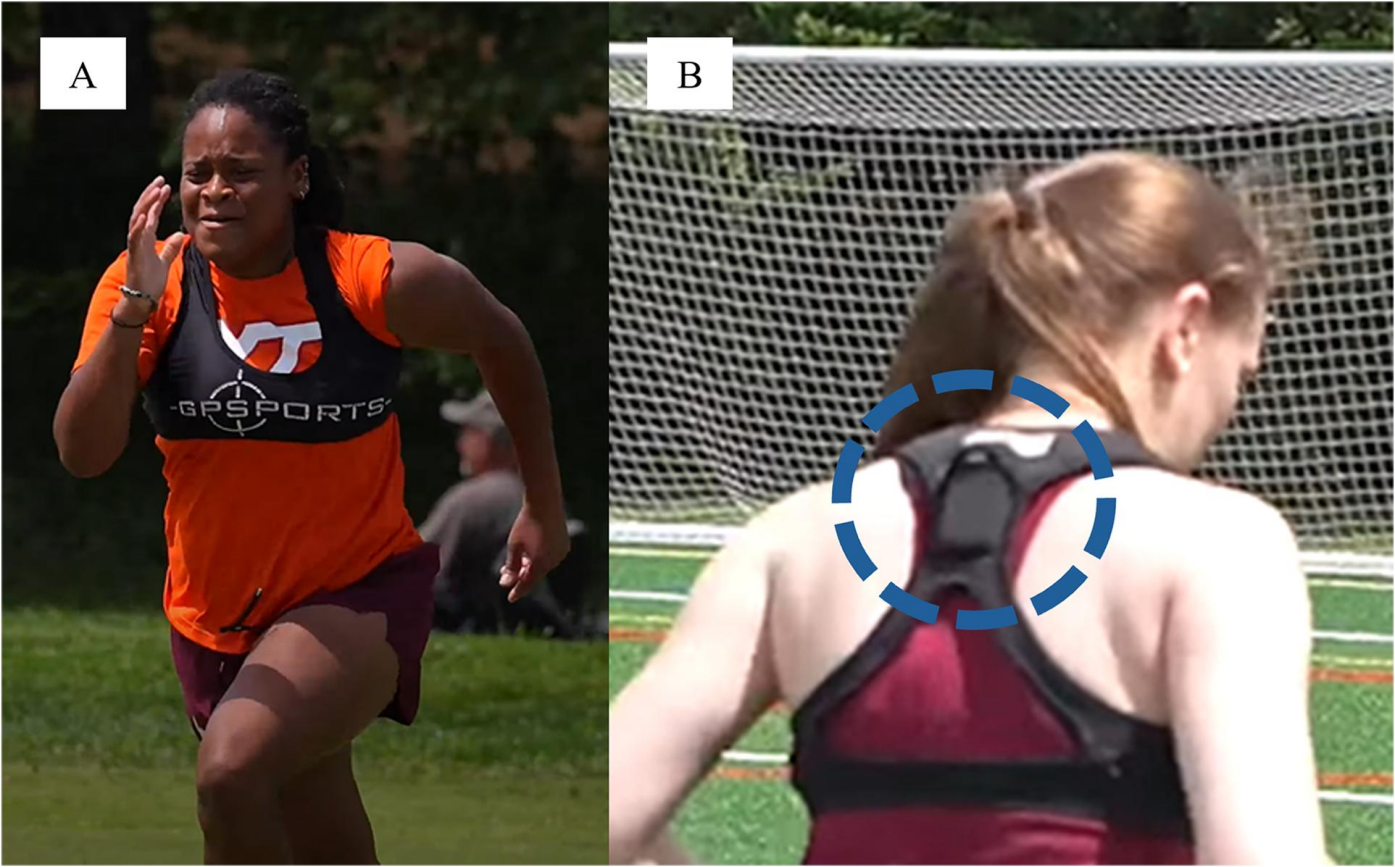
**(A)** Athlete wearing a STATSports APEX GPS/IMU unit secured within a vest during drill performance. **(B)** Close-up of GPS/IMU unit placement on the upper back (circled). The unit was used to measure running speed and trunk acceleration. Photograph credit: Virginia Tech Communications and Marketing.

Upon arrival, participants were fitted with standardized cleats (Nike Tiempo Legend 10 Pro; Nike, Beaverton, OR, USA) to control for footwear effects on performance (35). Participants were also equipped with a STATSports APEX GPS/IMU unit (18 Hz GPS, ±200 g 952 Hz tri-axial accelerometer, 952 Hz gyroscope, and 10 Hz magnetometer), which was used to measure running speed (km·h⁻¹) and peak trunk acceleration (m·s⁻²) (22, 23). This unit was secured to the participant's upper back using a manufacturer-provided vest (Figure 2).

**Figure 3:**
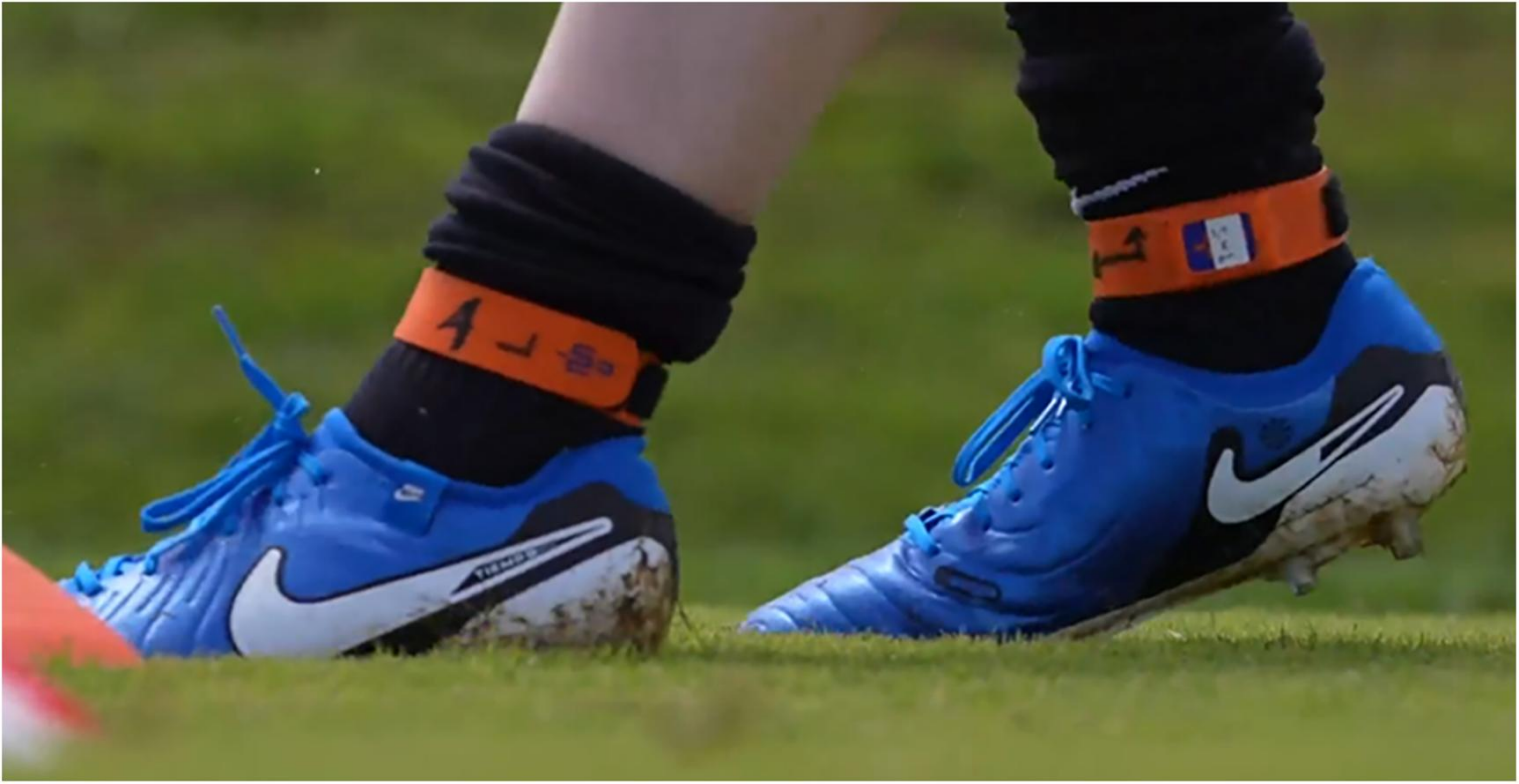
Standardized footwear (Nike Tiempo Legend 10 Pro cleats) and ankle-worn inertial measurement units (Vicon Blue Trident IMUs) used for data collection. IMUs were secured above the medial malleolus using orange straps to quantify lower-limb mechanical loading during athletic movements. Photograph credit: Virginia Tech Communications and Marketing.

Participants also wore a Blue Trident IMU (Vicon Motion Systems, Denver, CO) on each ankle, secured just above the medial malleolus using manufacturer-provided straps (Figure 3). Each unit featured two accelerometers (±16 g at 1,125 Hz and ±200 g at 1,600 Hz), a gyroscope (1,125 Hz), and a magnetometer (112 Hz), where g represents acceleration due to gravity (9.81 m·s⁻²). These devices have demonstrated high reliability in previous studies (29, 30) and were used to quantify lower-limb mechanical loading during movement. The following IMU-derived metrics were analyzed:

- Average intensity: the mean magnitude of impact-related accelerations (g) recorded at the lower limbs across all detected impacts.
- Bone stimulus: this metric, also referred to as daily load stimulus (DLS), was calculated by integrating peak tibial acceleration across loading cycles using an empirically derived relationship (36, 37). It reflects relative mechanical stimulus but does not represent total bone load, as muscular contributions are not included (38).
- Impact load per minute: the sum of impact-related accelerations (g) recorded at the lower limbs across all detected impacts, normalized per minute of activity (39). Total impact load was calculated as the sum of left and right limb contributions (39).

IMU-derived metrics have demonstrated inter-unit reliability in running-based tasks (intraclass correlation coefficients: 0.58–0.89 for impact load, 0.90–0.97 for bone stimulus, and ≥0.90 for most metrics) (19, 20). These sensors quantify external loading near the point of ground contact and are commonly used in athlete monitoring and rehabilitation contexts (19, 40, 41). Because drill duration and movement speed varied across conditions, impact load per minute was used to standardize comparisons across trials.

A within-subject pretest–posttest survey design was implemented (42), with surveys administered immediately before and after testing using Qualtrics (Qualtrics, Provo, UT, United States), accessed via QR codes on participants' mobile devices. Upon arrival at each field, participants completed a pre-performance survey assessing perceived surface quality (1–10 scale; 1 = lowest quality, 10 = highest quality), sleep duration and quality, muscle soreness, stress level, and mood. Each participant completed four pre-performance surveys (one per field). After completing drills within each designated area, participants completed a post-performance survey assessing their perception on: Rate of Perceived Exertion (RPE; 6–20 scale; 70), perceived surface firmness, surface quality, and perceived impact on performance (all 1–10 scales). Each participant completed eight post-performance surveys. Survey responses were used to integrate subjective perception with objective surface and biomechanical measurements, with sleep, soreness, stress, mood, and RPE were averaged across participants. Post-performance responses were analyzed at the area level, and differences between hard and soft areas were evaluated using one-way analysis of variance with Fisher's protected least significant difference (LSD) test (*α* = 0.05).

**Figure 4:**
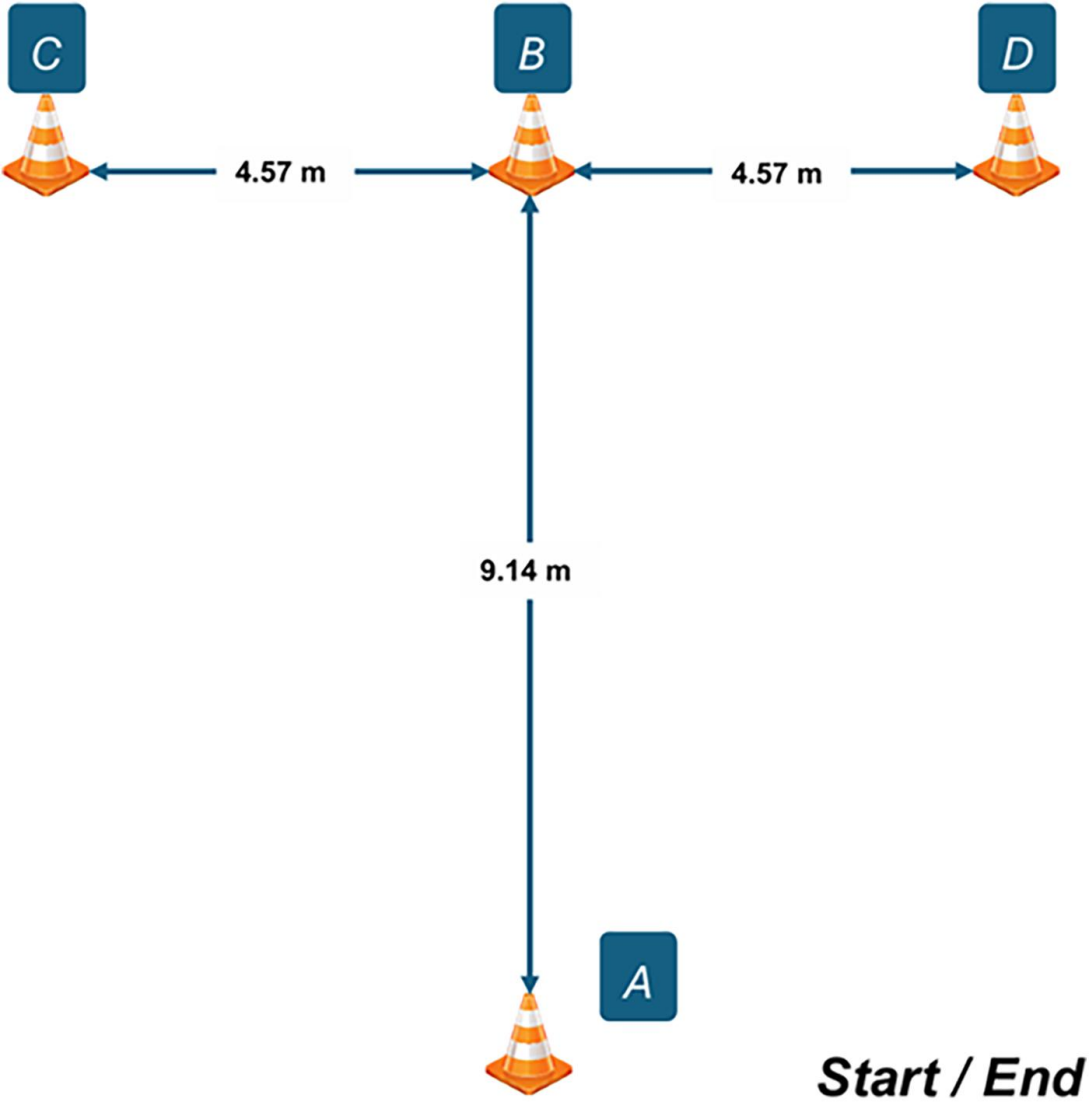
T-drill setup and movement pattern used to assess multidirectional speed and change-of-direction performance. The athlete sprinted from cone A to cone B, shuffled laterally to cone C, shuffled across to cone D, shuffled back to cone B, and backpedaled to the starting point at cone A. Distances between cones are indicated in the figure.

After completing the “pre-performance” survey, all athletes engaged in a ten-minute warm-up in a separate area from where the drills were to be performed. During this warm-up, the IMUs and STATSports GPS units were turned off, and therefore not recording data. Following the warm-up, participants were instructed to perform three repetitions of the following three drills within both the hard and soft areas of each field:

1. Drop landing: Participants stepped off a ∼40 cm plyometric platform (Yes4All, 3-in-1 wooden plyometric box) with their dominant foot, landing on both feet, and immediately performed a maximal vertical jump followed by a second landing. Three repetitions were completed with 15 s rest between trials. The platform was repositioned between participants, and participants stepped off different sides of the box between repetitions to minimize localized surface compaction (16).
2. T-drill (Figure 4): Participants sprinted forward from cone A to cone B, shuffled laterally to cone C, shuffled across to cone D, shuffled back to cone B, and backpedaled to cone A. Three repetitions were performed with 90 s rest between trials. Participants were instructed not to cross their feet during shuffling and to maintain a forward-facing trunk position. Touching the base of the cone was not required to minimize extraneous trunk rotation that could influence trunk acceleration data. Core positions were periodically adjusted to avoid localized wear.
3. Modified acceleration-deceleration: Participants sprinted 20 m at maximal speed, and then decelerated into a backward run back to the starting point. Three repetitions were performed with 90 s rest between trials (43). Starting locations were varied to limit localized surface compaction.

Drills were performed in this order to control for fatigue effects, progressing from least to most physically demanding. Participants were blinded to area classification. Testing order was consistent across days, and a 15-minute rest period was provided between fields.

### 2.5 Analysis of data during athlete involvement

Data from the STATSports GPS units were analyzed in JMP Pro 2024. Because each participant completed all field conditions, observations were not independent. To account for this repeated-measures structure, participant ID was included in the model, while field type, field usage, and hardness treated as fixed effects.

For the ankle-worn IMU units, three metrics relevant to loading and impact during athletic movements were analyzed: average intensity, impact load, and bone stimulus (20, 44). Analyses were conducted using a standard least squares model in JMP Pro 2024, with participant included in the model and field type, field usage, and hardness were treated as fixed effects.

Field type, field usage, and hardness were treated as fixed effects because the objective of this study was to compare the specific field conditions evaluated rather than make population-level inferences regarding all athletic fields. Participant ID was included in the model to account for repeated observations collected from the same athlete across field conditions. Linear mixed-effects models were considered; however, the relatively small sample size (*n* = 14 athletes), combined with the repeated-measures structure and limited number of field sites made estimation of more complex random-effects structures and interaction effects less reliable. Therefore, a standard least-squares approach including participant ID was selected to evaluate differences among the specific field conditions included in this study.

Two- and three-way interactions among fixed effects were initially evaluated; however, none were statistically significant and inclusion of these terms did not improve model fit. Therefore, a reduced model containing only main effects was retained. All IMU-derived variables were transformed as needed to approximate a normal distribution prior to analysis.

For each drill, the three repetitions performed by each participant were averaged within participant to generate a single representative value per drill. Because movement demands differed across drills, each drill was analyzed separately. Least squares means and associated standard errors were used to evaluate differences among fixed effects.

## 3 Results and discussion

### 3.1 Surface hardness data

**Figure 5:**
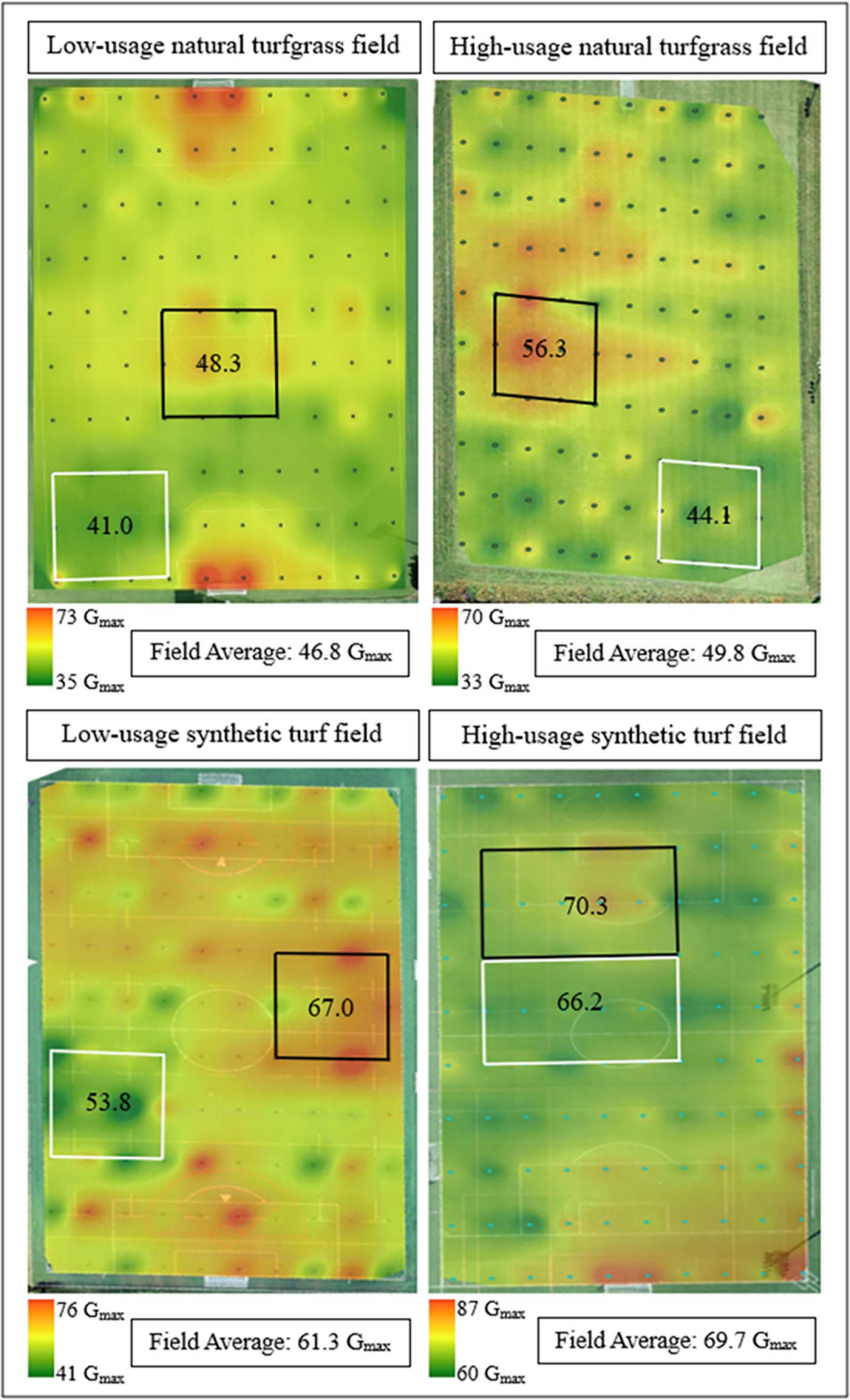
Spatial distribution of surface hardness (Gmax) across four athletic fields. Heatmaps illustrate within-field variability, with warmer colors indicating higher surface hardness and cooler colors indicating lower surface hardness. Black and white rectangles represent designated harder and softer areas, respectively, where athlete testing was conducted. Values within each rectangle indicate mean surface hardness for that area, and field average values are provided below each panel. Color scales indicate the range of surface hardness (green = softer and orange = harder) measured within each field.

Spatial heatmaps show variation of surface hardness within and across fields (Figure 5). Total field mean hardness differed significantly across all four fields (Table 2). Within-field hard-soft separation was most pronounced for the low-usage synthetic field and high-usage natural turfgrass field, whereas the softest field (low-usage natural turfgrass) and hardest field (high-usage synthetic turf) showed relatively small within-field differences at their respective extremes (Table 2).

**Table 2:** Mean surface hardness (Gmax) for each field, including whole-field averages and in designated hard and soft areas. Means followed by the same letter within a column are not significantly different according to Fisher's protected least significant difference (LSD) test at *α* = 0.05.

Field	Whole-Field Mean	Hard Area Mean	Soft Area Mean
Low-usage natural turfgrass field	46.8 d	48.3 c	41.0 c
High-usage natural turfgrass field	49.8 c	56.3 b	44.1 c
Low-usage synthetic turf field	61.3 b	67.0 a	53.8 b
High-usage synthetic turf field	69.7 a	70.3 a	66.2 a

Across field types, synthetic turf surfaces were significantly harder than natural turfgrass (*p* < 0.001; Table 2). From a biomechanical perspective, increased surface firmness reduces surface deformation and attenuation, thereby increasing the proportion of impact forces transmitted to the lower limbs during movement (7, 8, 45). Neither synthetic field included a shock pad, which is designed to enhance impact attenuation and better replicate the cushioning characteristics of natural turfgrass systems (45, 46). In addition, synthetic turf can become progressively firmer as athlete traffic compacts and/or displaces infill and reduces the surface's capacity to dissipate impact energy (15, 47). Although maintenance practices like decompaction and infill replenishment can reduce this hardness (48, 49), synthetic turf lacks the biological and structural cushioning properties of natural turfgrass (14, 50).

In contrast, the natural turfgrass fields in this study exhibited lower surface hardness values, which may be related to the high soil moisture levels recorded in both the hard and soft areas (Table 3). According to FIFA guidelines, soil moisture exceeding 35% volumetric water content is classified as extremely wet (50), which was the case for the tested natural turfgrass fields. Previous research consistently shows that increasing soil moisture content decreases surface hardness by allowing the soil to deform more readily under impact (11, 51). The relatively high soil moisture levels observed during sampling are therefore consistent with the softer surface hardness of the natural turfgrass fields. Additionally, when examining total field averages within natural turfgrass and synthetic turf fields, the high-usage fields were significantly harder than the low-usage fields (Table 2). Increased usage or traffic has been shown to compact both soil and synthetic turf infill, leading to higher surface hardness over time (9, 15, 49). All surface hardness measurements recorded in this study fell below 100 Gmax, meeting acceptable quality criteria under the FIFA natural-pitch rating system (50); however, the observed variability indicates that athletes may still experience different surface responses within and across fields.

**Table 3:** Mean rotational resistance (all surfaces), soil moisture and thatch depth (natural turfgrass), and infill depth (synthetic turf) measured within designated hard and soft areas of each field. Values represent area means, and the difference (Hard−Soft) is included to illustrate within-field variability.

Field	Metric	Hard Area Mean	Soft Area Mean	Hard−Soft
Low-usage natural turfgrass	Rotational resistance (N m)	32.9	30.4	+2.5
	Soil moisture (%)	40.9	39.4	+1.5
	Thatch depth (mm)	27.4	40.9	−13.5
High-usage natural turfgrass	Rotational resistance (N m)	35.1	33.2	+1.9
	Soil moisture (%)	35.8	42.4	−6.6
	Thatch depth (mm)	11.1	25.1	−14.0
Low-usage synthetic turf	Rotational resistance (N m)	25.6	25.8	−0.2
	Infill depth (mm)	39.7	42.3	−2.6
High-usage synthetic turf	Rotational resistance (N m)	22.3	24.2	−1.9
	Infill depth (mm)	31.3	33.5	−2.2

Studies in the literature demonstrate that such variability can influence the way athletes move. Sleat et al. (8) compared athlete movement patterns across two natural turfgrass fields differing in hardness and found that the softer field promoted more high-intensity shuffling and a greater frequency of movements such as running, dribbling, and sharp directional changes. Although overall match events were similar, several movement categories differed between surfaces, suggesting that hardness can shape how athletes interact with the field. Forrester et al. (33) further showed that surface hardness can vary substantially across synthetic turf fields, with spatial variation sometimes exceeding temporal variation. Their results suggest the magnitude of within-field variation in surface hardness may be detectable by players; however, the specific effects on athlete biomechanics and perception remain unclear and warrant further investigation. Overall, the variability in surface hardness observed in this study suggests that athletes may encounter different surface responses within the same field, which can influence how they load and interact with the surface during movement.

### 3.2 Field variability

Resulting rotational resistance, thatch depth (natural turfgrass fields only), soil moisture (natural turfgrass fields only), and infill depth (synthetic turf fields only) measured within hard and soft areas across all fields are presented in Table 3. Rotational resistance differed significantly by field type (*p* < 0.001) and exhibited significant field type × field usage (*p* < 0.001) and field type × hard/soft area interactions (*p* = 0.001), indicating that patterns of rotational resistance varied across surface types and usage levels. In contrast, field usage alone and hard versus soft area alone were not significant main effects (*p* > 0.13), suggesting within-field differences depended on surface type. Across the natural turfgrass fields evaluated in this study, rotational resistance remained within the acceptable range defined by the FIFA natural-pitch rating system (25–50 N m; 51). Although absolute differences between hard and soft areas were modest, these results indicate that rotational response varied spatially within fields, which may influence athlete movement during cutting and turning tasks (52).

In the natural turfgrass fields, thatch depth differed significantly by field usage (*p* < 0.001) and between hard and soft areas within the field (*p* < 0.001), indicating both between-field and within-field variability among the natural turfgrass fields (Table 3). The low-usage natural turfgrass field exhibited greater thatch accumulation than the high-usage field, despite routine aeration (averages two hollow-tine aeration events in summer and two solid-tine aeration events during the fall soccer season) and periodic vertical mowing. This is likely due to its earlier establishment date (2008–2009), which provided a longer period for thatch development, as well as increased fertility inputs and associated mowing frequency on the low-usage field. In contrast, the high-usage natural turfgrass field had recently been established (2023; a year prior to data collection), explaining its shallower thatch depth due to lower organic matter accumulation at the time of this study. Because greater thatch depths can increase surface cushioning, these differences also align with the surface hardness patterns observed between the two natural turfgrass fields (53, 54).

Soil moisture differed between within-field hard and soft areas, with the effects test indicating a significant main effect (*p* < 0.01) and a significant interaction between field usage and hard/soft area (*p* < 0.001). In the low-usage natural turfgrass field, the hard area exhibited slightly higher soil moisture than the soft area (+1.5%), whereas the opposite pattern was observed in the high-usage natural turfgrass field (−6.6%; Table 3). These results reflect the inherently variable nature of soil moisture and suggest that localized soil moisture conditions differed between and within fields. Given the high temporal and spatial variability of soil moisture (12, 55), these measurements were primarily used to contextualize observed differences in surface hardness rather than as a primary driver of surface performance.

In the synthetic turf fields, infill depth differed significantly between usage levels and between hard and soft areas (*p* < 0.001). Specifically, the high-usage synthetic field exhibited shallower infill, consistent with previous findings that infill depth decreases as fields age and receive more traffic [(15); Table 3]]. Reduced infill depth corresponded with the higher surface hardness measured on that field, reflecting the established inverse relationship between infill depth and surface hardness (47, 49). Within synthetic fields, soft areas contained slightly greater infill depths than hard areas, consistent with patterns of infill redistribution resulting from foot traffic and maintenance practices (56). Dickson et al. (15) reported that infill depths ≥35 mm are associated with more than 95% of surface hardness measurements remaining below 100 Gmax. In the present study, the hard area within the high-usage synthetic field had a mean infill depth below this threshold, indicating localized infill loss that may warrant maintenance. However, surface hardness in this area remained below 100 Gmax, and the findings of Dickson et al. (15) may not be directly applicable to the field conditions evaluated in this study.

Overall, rotational resistance, soil moisture, thatch depth, and infill depth surface characteristics exhibited variability across fields, as well as between hard and soft areas within fields. These differences provide context for the surface hardness patterns discussed earlier and highlight the spatial complexity of athlete–surface interactions (4, 5, 30).

### 3.3 Athlete performance data

No statistically significant differences in running speed were observed across participants, field types, field usage, or hardness for either the T-drill or modified acceleration–deceleration drill. Running speed is known to influence lower-limb mechanical loading, with higher speeds leading to increased impact forces (38, 57). The absence of speed differences indicates that observed IMU responses were not confounded by variation in movement velocity across conditions.

Analysis of average peak trunk acceleration during the drop landing revealed no statistically significant differences across field type, usage, or hardness. Trunk acceleration is commonly used as an indicator of landing load and ground reaction forces and is influenced by trunk posture and control during landing (58). Prior research has shown that reduced trunk flexion or increased lateral bending can influence knee loading patterns that have been associated with ACL injury mechanisms (26, 27, 59, 60), and that female athletes often exhibit greater trunk acceleration during landings (29, 61, 62). Although trunk acceleration was included due to its relevance to landing mechanics, it was not sensitive to the surface conditions evaluated in this study.

Effects tests indicated significant main effects of field type, field usage, and hardness on IMU metrics, with significant values bolded (Table 4). Individual participant variation accounted for the largest portion of variability for all three IMU metrics; however, participant identity was included in the model to account for the repeated-measures structure (Table 4). For clarity, mean values across all athletes are presented to summarize overall trends (Table 5). Significance testing was conducted on transformed data, with transformations applied as needed for each variable to satisfy normality assumptions, and athletes serving as their own controls. Values were subsequently back-transformed to the original units for reporting; therefore, differences in means may appear numerically small despite being statistically significant, reflecting consistent within-athlete differences with small standard errors.

**Table 4:** *P*-values from effects tests evaluating main effects of field type, field usage, and within-field hardness across drills (drop landing, T-drill, and modified acceleration–deceleration) and IMU-derived metrics (average intensity, bone stimulus, and impact load per minute).

Metric	Average Intensity (g)			Bone Stimulus (−)			Impact Load Per Minute (g)		
Drill	Drop Landing	T-Drill	Modified Accel–Decel	Drop Landing	T-Drill	Modified Accel–Decel	Drop Landing	T-Drill	Modified Accel–Decel
Participant ID	**<0.01**	**<0**.**01**	**<0.01**	**<0.01**	**<0.01**	**<0.01**	**<0**.**01**	**<0**.**01**	**<0**.**01**
Field Type [Participant ID]	**<0.01**	**<0**.**01**	**<0.01**	**<0.05**	**<0.01**	NS	**<0**.**01**	**<0**.**01**	**<0**.**01**
Field Usage [Participant ID]	NS	**<0**.**01**	NS	NS	NS	NS	**<0**.**01**	**<0**.**01**	**<0**.**01**
Hardness [Participant ID]	NS	**<0**.**01**	**<0.01**	NS	NS	NS	**0**.**0331**	**<0**.**01**	NS

Bold values indicate statistical significance at *α* = 0.05. NS indicates no significant effect. Participant ID included in the model to account for repeated measures.

**Table 5:** Back-transformed means (± SE) for IMU-derived metrics (average intensity, bone stimulus, and impact load per minute) across drills (drop landing, T-drill, and modified acceleration–deceleration) by field type, field usage, and hardness.

		Field Type		Field Usage		Hardness	
Metric	Drill	Natural Turfgrass	Synthetic Turf	Low Usage	High Usage	Soft	Hard
Average Intensity (g)	Drop Landing	19.7 ± 1.9	22.7 ± 1.8	NS	NS	NS	NS
	T-Drill	15.8 ± 1.2	18.1 ± 1.2	16.5 ± 1.2	18.1 ± 1.2	17.05 ± 1.2	17.4 ± 1.2
	Mod. Accel-Decel	17.7 ± 1.2	21.4 ± 1.1	NS	NS	18.9 ± 3.8	20.4 ± 4.3
Bone Stimulus (−)	Drop Landing	137.1 ± 1.9	138.3 ± 1.8	NS	NS	NS	NS
	T-Drill	150.3 ± 2.2	163.9 ± 2.1	NS	NS	NS	NS
	Mod. Accel-Decel	NS	NS	NS	NS	NS	NS
Impact Load Per Minute (g)	Drop Landing	5,072.0 ± 440.9	5,919.1 ± 424.9	5,949.5 ± 408.3	5,117.8 ± 408.3	5,407.1 ± 410.7	5,638.8 ± 410.7
	T-Drill	3,218.1 ± 329.1	3,658.7 ± 317.1	3,641.3 ± 341.0	3,448.0 ± 341.0	3,374.7 ± 330.1	3,682.3 ± 330.1
	Mod. Accel-Decel	4,149.6 ± 388.3	4,623.3 ± 372.2	4,813.2 ± 403.5	4,127.8 ± 403.5	NS	NS

NS indicates no significant effect (*p* ≥ 0.05).

Field type significantly affected all three IMU metrics (Table 4). Across drills, synthetic turf consistently resulted in higher average intensity, higher bone stimulus (except in the modified acceleration–deceleration drill), and higher impact load per minute compared to natural turfgrass (Table 5). These patterns are consistent with the greater surface hardness measured on synthetic turf fields (Table 2).

Field usage also influenced several IMU metrics. In the T-drill, high-usage fields produced higher average intensity values than low-usage fields, whereas for impact load per minute the pattern was reversed, with low-usage fields producing higher loads across all drills (Table 5). These contrasting responses likely reflect differences in surface condition associated with usage and suggest that athletes may adapt movement strategies based on perceived surface properties (63, 64).

Hardness effects followed a consistent direction across drills. Hard areas on each field produced higher average intensity in the T-drill and modified acceleration–deceleration drill, and higher impact load per minute in both the drop-landing and T-drill (Table 5). Although the magnitude of these differences was modest, the consistent direction of these effects supports a relationship between surface firmness and lower-limb mechanical loading.

Overall, harder surfaces generally produced higher IMU-derived loading in this study across field type, field usage, and hardness categories. While these findings demonstrate associations between surface characteristics and IMU-derived loading metrics, the present study did not assess athlete injury occurrence or long-term health outcomes. Therefore, the observed differences in mechanical loading should not be interpreted as direct indicators of injury risk. Synthetic turf fields, which were significantly firmer than natural turfgrass surfaces (Table 2), generated higher average intensity, bone stimulus, and impact load per minute in nearly all drill–metric combinations (Tables 4, 5). The synthetic turf surfaces produced average intensity values within the IMU Step “high-intensity” classification range (21.5–26.7 g); however, this classification is descriptive and does not imply biological thresholds or injury risk. Rather, these findings illustrate how typical differences in surface properties can translate into measurable differences in lower-limb loading during athletic movements.

### 3.4 Athlete perceptions

**Figure 6:**
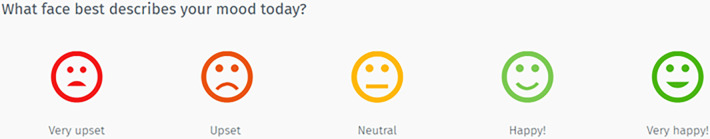
Visual mood scale used in the pre-performance survey, where participants selected the face that best represented their current mood prior to testing on each field. The scale ranges from very upset to very happy. Adapted from QuestionPro (virginiatech.questionpro.com).

Prior to testing on each field, athletes completed a pre-performance survey assessing perceived surface quality, as well as their sleep quality, soreness levels, stress levels, and mood [Table 6; Figure 6 (65)]. These measures were used to characterize athlete condition prior to testing, as these factors may influence performance or perception. Following drills in each area of each field, athletes completed a post-performance survey evaluating perceived exertion (66, 67), surface firmness, surface quality, and the perceived impact of the surface on their performance.

**Table 6:** Athlete perceptions of surface quality (1–10 scale, 10 highest quality) collected before and after testing. Means followed by the same letter within a column are not significantly different according to Fisher's protected least significant difference (LSD) test at *α* = 0.05.

Field	Pre-Performance Rating	Post-Performance Rating
Low-usage natural turfgrass field	8.78 a	8.57 a
High-usage natural turfgrass field	4.42 c	4.14 d
Low-usage synthetic turf field	8.14 a	7.53 b
High-usage synthetic turf field	7.07 b	6.71 c

Across all participants, athletes reported an average of 7.46 h of sleep with moderate sleep quality (6.69 out of 10) and relatively low soreness (4.67 out of 10), most commonly in the quadriceps, hamstrings, and hip flexors. Stress levels were also low on average (4.47 out of 10), and 71.43% of athletes selected the “Happy!” mood option (Figure 6). Post-performance RPE averaged 15.76, indicating substantial effort during the drills.

Clear patterns emerged across fields (Tables 6, 7). The low-usage natural turfgrass field consistently received the highest quality ratings both before and after drill performance (although statistically similar to the low-usage synthetic field before drill performance). It also earned the highest surface-impact ratings, suggesting that athletes felt this field supported their performance more positively than the other surfaces (Table 7). In contrast, the high-usage natural turfgrass field received the lowest ratings across nearly all categories. These lower ratings likely reflect reduced surface quality associated with heavier use, although specific causes were not directly assessed. The two synthetic turf fields generally received intermediate ratings, with perceptions of both exhibiting better quality than the high-usage natural turfgrass field. The low-usage synthetic turf field was perceived to be similar to the low-usage natural turfgrass field prior to testing, but of lower quality following the participants completing the drills. Across both pre- and post-performance evaluations, low-usage fields were generally rated higher than high-usage fields. This pattern suggests that athletes perceived the synthetic turf surfaces more favorably than the heavily worn natural turfgrass surface, but not to the same degree as the well-maintained, low-usage natural turfgrass field. It also suggests that athletes could discern between the low- and high-usage synthetic turf fields used in this study.

**Table 7:** Post-performance survey responses for each field, pooled across hard and soft areas due to no significant differences between areas. Ratings were assessed on 1–10 scales for perceived surface firmness (1 = very soft, 10 = very hard), perceived surface impact (1 = negative impact on performance, 10 = positive impact), and perceived surface quality (1 = poor quality, 10 = excellent quality). Means followed by the same letter within a column are not significantly different according to Fisher's protected least significant difference (LSD) test at *α* = 0.05.

Field	Mean Rating (Hard and Soft Areas Combined)		
	Firmness	Surface Impact	Surface Quality
Low-usage natural turfgrass field	6.17 a	7.39 a	8.57 a
High-usage natural turfgrass field	4.17 b	4.75 c	4.14 d
Low-usage synthetic turf field	5.85 a	6.28 b	7.53 b
High-usage synthetic turf field	6.42 a	5.32 c	6.71 c

The low-usage natural turfgrass field, the consistently highest-rated surface (Table 6), also had the lowest surface hardness among the four fields (Table 2). This aligns with results from Straw et al. (30), who found that athletes tended to prefer areas with lower surface hardness and higher objective turfgrass quality values as measured by normalized difference vegetation index (NDVI), identifying these areas as the “best” within natural turfgrass fields. These results further support the relationship between lower surface hardness and more favorable athlete perception. Athlete feedback provides insight into how field-specific conditions are experienced during performance. In this study, surfaces with lower hardness and better overall quality tended to receive more favorable ratings, whereas surfaces with more frequent use and reduced quality received lower ratings. These perception-based results complement the objective surface measurements and provide a more complete understanding of how different field conditions may influence athlete satisfaction and perceived performance.

Taken together, the findings of this study suggest that within- and between-field variability in surface characteristics may influence athlete responses across multiple domains. Areas exhibiting greater surface hardness generally corresponded with increased IMU-derived loading metrics, while athletes also reported differences in perceived surface quality and impact of surface conditions on performance. Although objective performance measures showed fewer consistent differences among field conditions, the combined results indicate that athletes experienced meaningful differences in localized surface conditions. Although objective loading responses and subjective perceptions were not always directly aligned, these findings suggest that surface variability may influence mechanical loading, perceived performance, and overall athlete experience. These findings highlight the value of integrating objective surface measurements, athlete-derived biomechanical metrics, and perceptual assessments when evaluating athletic fields. From a practical perspective, routine monitoring of surface characteristics may help identify localized areas that differ from field-wide averages and may therefore be experienced differently by athletes. Future research should examine these relationships across a broader range of fields, environmental conditions, and athlete populations.

### 3.5 Limitations

This study represents a small sample size (14 participants), which restricted the complexity of statistical models that could be fitted. Due to the relatively small sample size, statistical power to detect smaller effect sizes may have been limited, and non-significant findings should be interpreted with caution. No *a priori* power analysis was conducted to determine sample size. The study may have been underpowered for detecting smaller effects, so non-significant findings should not necessarily be interpreted as evidence of no effect. Although interactions among the fixed effects field type, field usage, and hardness were evaluated, the relatively small sample size may have limited statistical power to detect potentially meaningful interaction effects. Additionally, while Participant ID was included in the model to account for repeated observations from the same athlete, a linear mixed-effects framework would provide a more comprehensive approach for modeling repeated measures and nested sources of variability. The limited number of participants and field sites restricted the feasibility of fitting more complex models. In addition to participant sample size, the study included only four field sites, which limits the ability to generalize findings across a broader range of athletic surfaces and management conditions. While inclusion of additional field sites may have improved generalizability, testing more fields within the same experimental session could have introduced participant fatigue as a confounding variable. For both the STATSports GPS and ankle IMU data, field type, field usage, and hardness were modeled as fixed effects to allow for direct comparisons of means across field conditions. As such, the findings of this study are not generalizable to all athletic fields or to all athletes.

The results are specific to the fields tested and the participant group evaluated in this study. Testing was conducted during afternoon hours; however, environmental conditions such as temperature, humidity, and weather were not formally recorded and therefore could not be evaluated as potential sources of variation between testing days. As a result, the potential influence of environmental conditions on surface characteristics and athlete responses cannot be excluded. Surface characteristics such as soil moisture and hardness are known to vary temporally, and measurements in this study represent a single time point rather than seasonal or multi-day variability. Caution should be taken when extrapolating these results beyond the sampled fields or athlete population.

Hard and soft areas were defined relative to each field rather than using absolute thresholds, which limits direct comparison of hardness classifications across fields. Additionally, the order of field testing was not randomized across surface types. All athletes completed testing on the synthetic turf fields on the first day and the natural turfgrass fields on the second day, with field order remaining consistent across participant groups. Consequently, differences between surface types may have been influenced by factors other than surface characteristics alone, including day-to-day environmental variation, residual fatigue or recovery effects between testing days, and potential learning or familiarization effects associated with repeated completion of the testing protocol. Although participants completed standardized warm-up procedures and testing schedules on both days, these potential sources of bias cannot be fully separated from the observed surface-type effects and should be considered when interpreting the results.

Finally, athlete performance was assessed using controlled drill-based movements rather than in-game conditions, which may not fully capture the complexity and variability of sport-specific movement patterns. As a result, the observed athlete performance and perception responses are specific to the testing protocol employed in this study and should be interpreted within that context.

## 4 Conclusion

This study demonstrated substantial variation in surface hardness both within and between fields. The synthetic turf fields tested were generally harder than the natural turfgrass fields, and high-usage fields, regardless of surface type, tended to be harder than low-usage fields. These differences enabled targeted evaluation of relatively hard and soft areas within each field, which revealed additional variability in rotational resistance, soil moisture, thatch depth, and infill depth. Together, these results highlight that spatial variability exists across both natural turfgrass and synthetic turf athletic fields. Interpolated maps of surface hardness were used to guide athlete testing locations for performance and perceptions assessments.

IMU-derived loading metrics of human athlete participants aligned with these surface characteristics. Harder surfaces, particularly the synthetic turf fields and the harder areas within each field, were associated with greater average intensity, bone stimulus, and impact load per minute during athletic movements. High-usage fields also showed elevated values during some drills compared with low-usage fields. These findings demonstrate that increased surface hardness was associated with greater lower-limb mechanical loading under the conditions evaluated in this study. While these patterns may have implications, this study does not establish injury thresholds or causal relationships between surface characteristics and injury outcomes. Additional research is needed to determine whether how IMU-derived loading metrics relate to injury risk over time.

Athlete perception data further illustrated how field conditions influenced the participant experience. The well-maintained, low-usage natural turfgrass field received the highest ratings for quality and surface impact, whereas the high-usage natural turfgrass field received the lowest ratings across survey categories. Both synthetic turf fields were rated between these two natural surfaces, suggesting that overall field condition, rather than surface type alone, shaped athlete perceptions of surface quality and performance impact. These findings emphasize the importance of considering both objective surface measurements and athlete perception when evaluating athletic field performance.

## Data Availability

The raw data supporting the conclusions of this article will be made available by the authors, without undue reservation.
